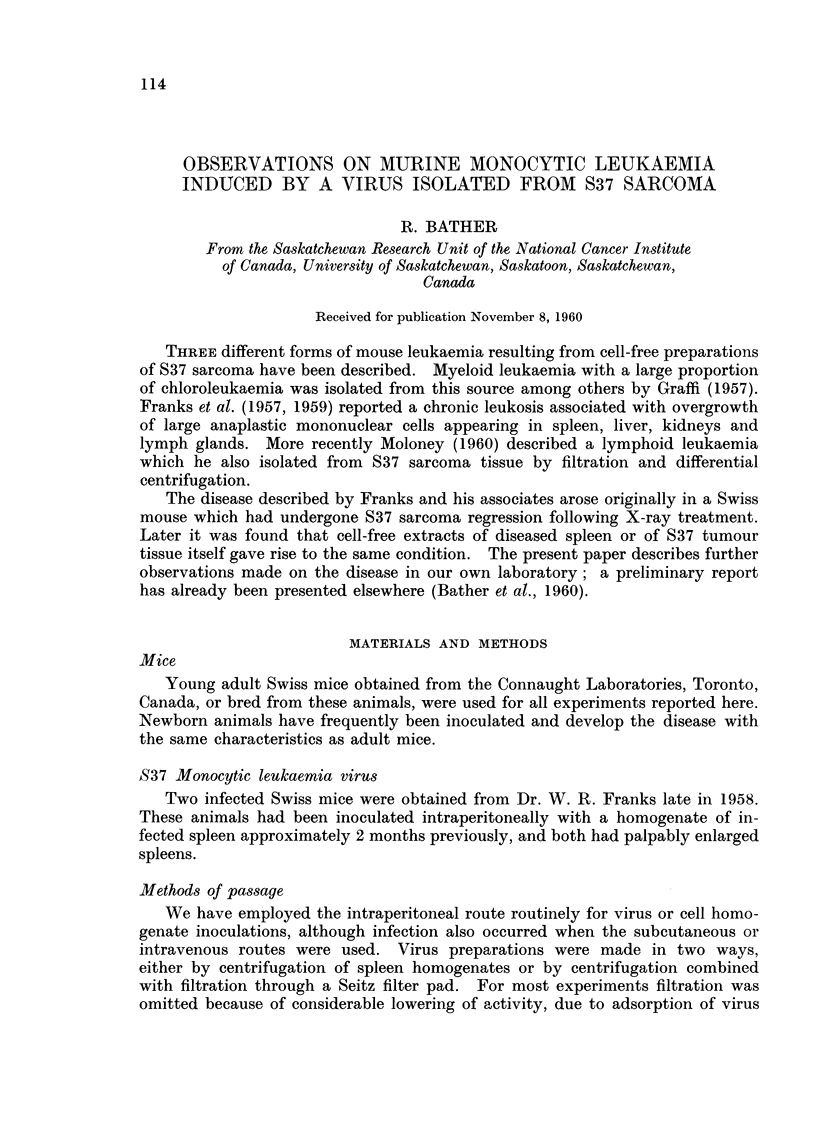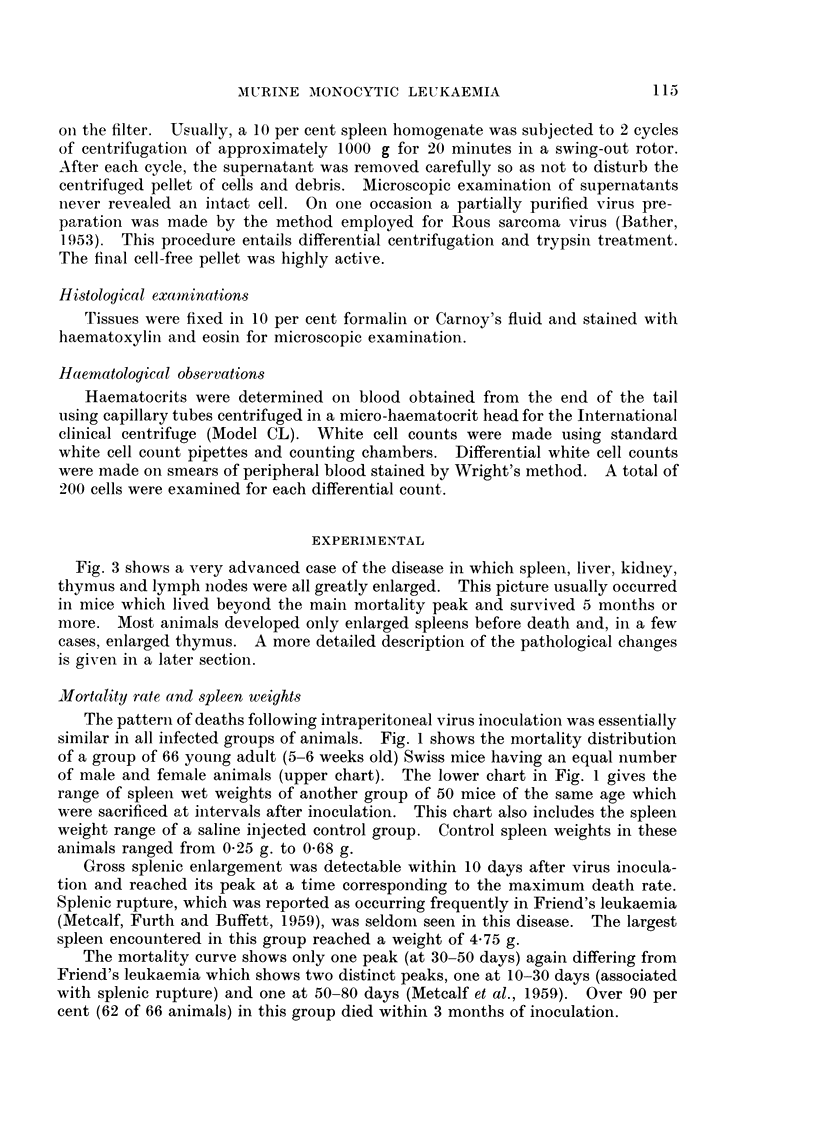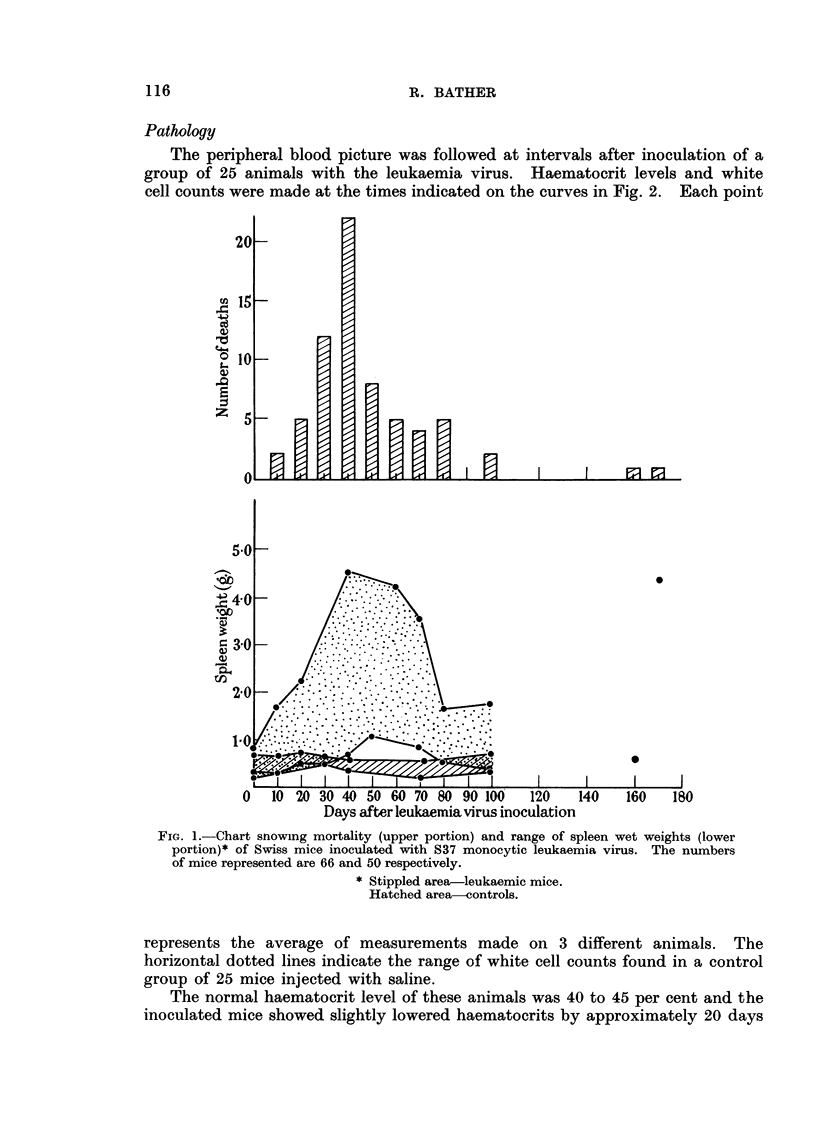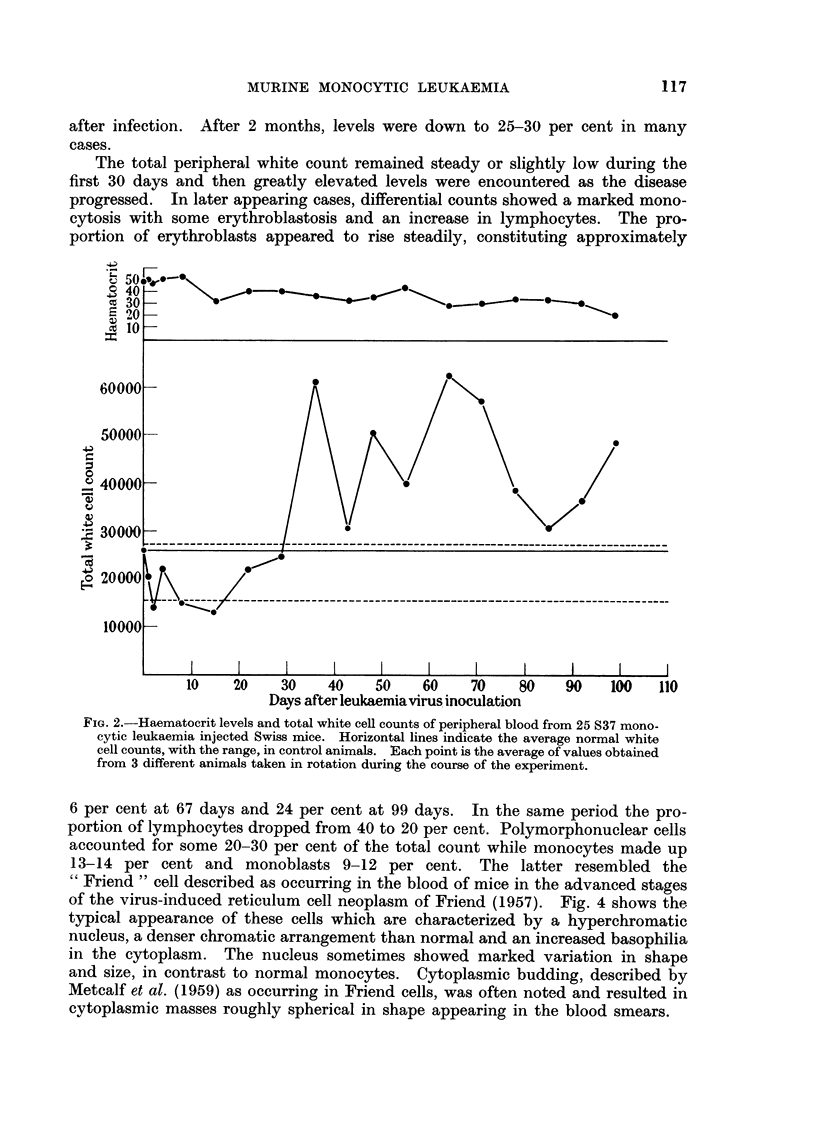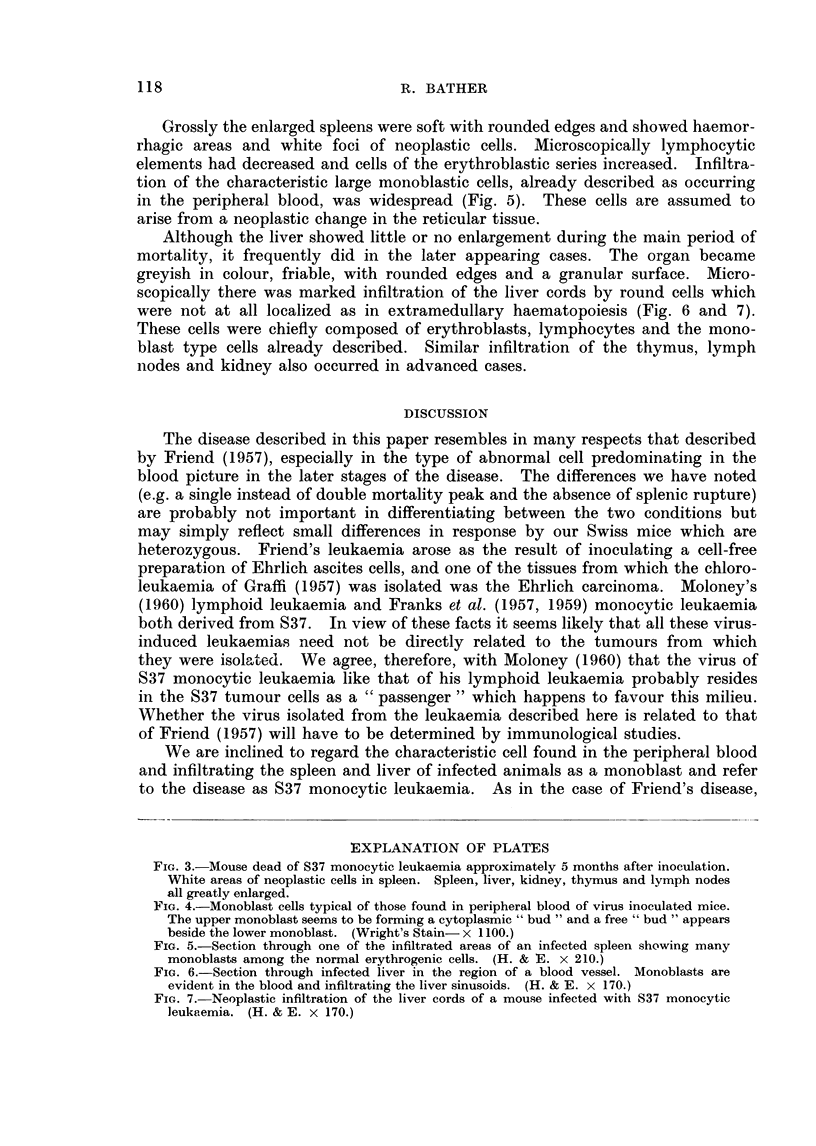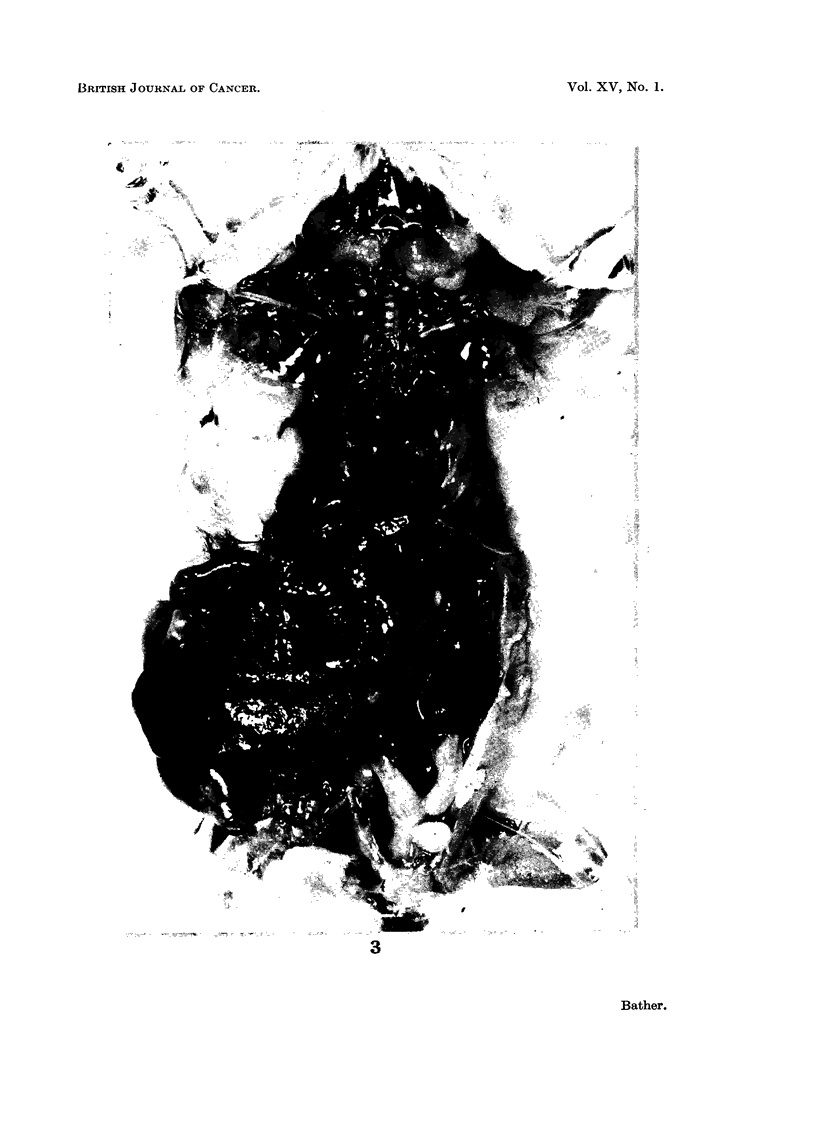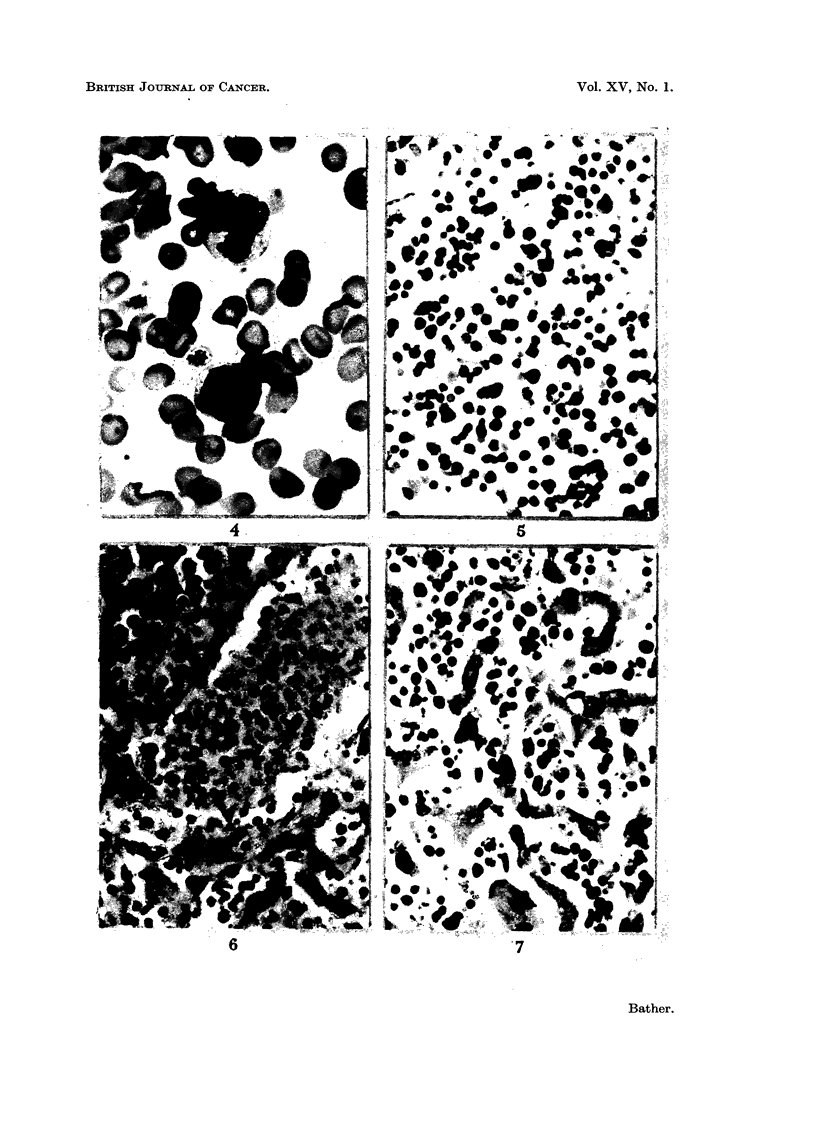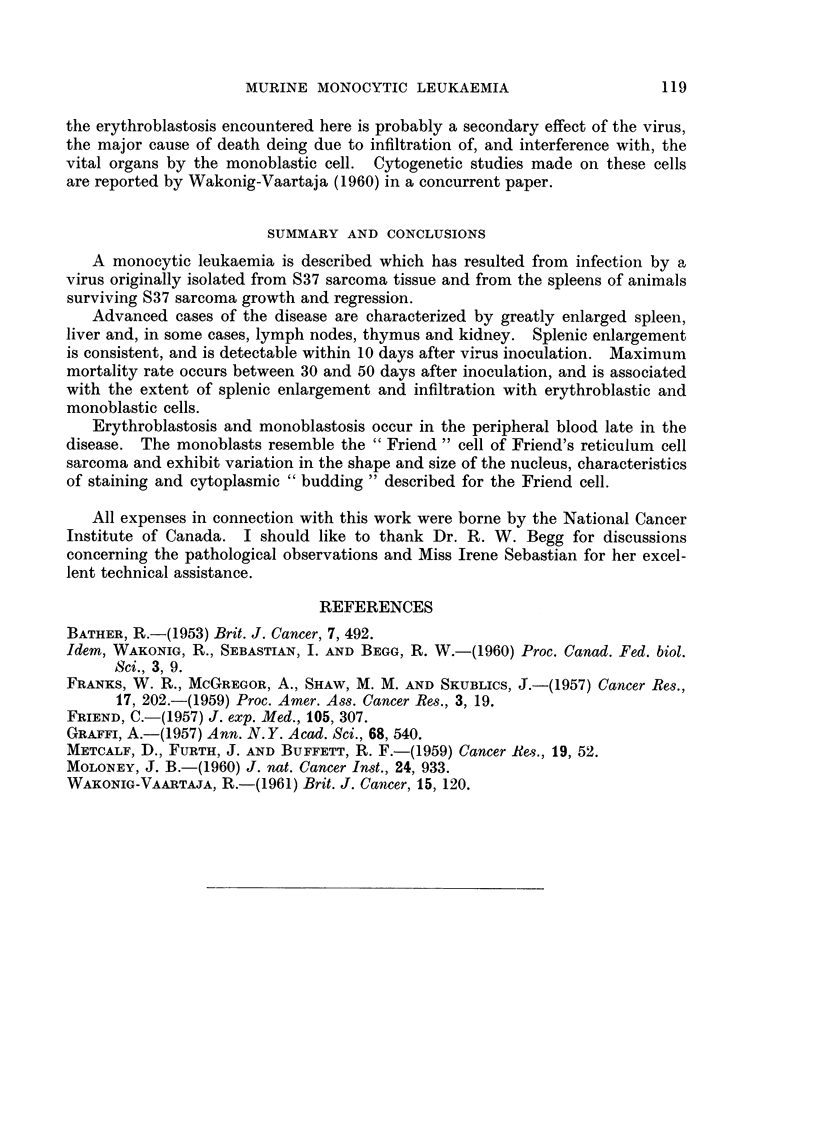# Observations on Murine Monocytic Leukaemia Induced by a Virus Isolated From S37 Sarcoma

**DOI:** 10.1038/bjc.1961.12

**Published:** 1961-03

**Authors:** R. Bather

## Abstract

**Images:**


### OBSERVATIONS ON MURINE MONOCYTIC LEUKAEMIA INDUCED BY A VIRUS ISOLATED FROM S37 SARCOMA

R. BATHER

*From the Saskatchewan Research Unit of the National Cancer Institute of Canada, University of Saskatchewan, Saskatoon, Saskatchewan, Canada*

Received for publication November 8, 1960

THREE different forms of mouse leukaemia resulting from cell-free preparations of S37 sarcoma have been described. Myeloid leukaemia with a large proportion of chloroleukaemia was isolated from this source among others by Graffi (1957). Franks *et al.* (1957, 1959) reported a chronic leukosis associated with overgrowth of large anaplastic mononuclear cells appearing in spleen, liver, kidneys and lymph glands. More recently Moloney (1960) described a lymphoid leukaemia which he also isolated from S37 sarcoma tissue by filtration and differential centrifugation.

The disease described by Franks and his associates arose originally in a Swiss mouse which had undergone S37 sarcoma regression following X-ray treatment. Later it was found that cell-free extracts of diseased spleen or of S37 tumour tissue itself gave rise to the same condition. The present paper describes further observations made on the disease in our own laboratory ; a preliminary report has already been presented elsewhere (Bather *et al.*, 1960).

### MATERIALS AND METHODS

### *Mice*

Young adult Swiss mice obtained from the Connaught Laboratories, Toronto, Canada, or bred from these animals, were used for all experiments reported here. Newborn animals have frequently been inoculated and develop the disease with the same characteristics as adult mice.

### *S37 Monocytic leukaemia virus*

Two infected Swiss mice were obtained from Dr. W. R. Franks late in 1958. These animals had been inoculated intraperitoneally with a homogenate of infected spleen approximately 2 months previously, and both had palpably enlarged spleens.

### *Methods of passage*

We have employed the intraperitoneal route routinely for virus or cell homogenate inoculations, although infection also occurred when the subcutaneous or intravenous routes were used. Virus preparations were made in two ways, either by centrifugation of spleen homogenates or by centrifugation combined with filtration through a Seitz filter pad. For most experiments filtration was omitted because of considerable lowering of activity, due to adsorption of virus on the filter. Usually, a 10 per cent spleen homogenate was subjected to 2 cycles of centrifugation of approximately 1000 **g** for 20 minutes in a swing-out rotor. After each cycle, the supernatant was removed carefully so as not to disturb the centrifuged pellet of cells and debris. Microscopic examination of supernatants never revealed an intact cell. On one occasion a partially purified virus preparation was made by the method employed for Rous sarcoma virus (Bather, 1953). This procedure entails differential centrifugation and trypsin treatment. The final cell-free pellet was highly active.

*Histological examinations*

Tissues were fixed in 10 per cent formalin or Carnoy's fluid and stained with haematoxylin and eosin for microscopic examination.

*Haematological observations*

Haematocrits were determined on blood obtained from the end of the tail using capillary tubes centrifuged in a micro-haematocrit head for the International clinical centrifuge (Model CL). White cell counts were made using standard white cell count pipettes and counting chambers. Differential white cell counts were made on smears of peripheral blood stained by Wright's method. A total of 200 cells were examined for each differential count.

### EXPERIMENTAL

Fig. 3 shows a very advanced case of the disease in which spleen, liver, kidney, thymus and lymph nodes were all greatly enlarged. This picture usually occurred in mice which lived beyond the main mortality peak and survived 5 months or more. Most animals developed only enlarged spleens before death and, in a few cases, enlarged thymus. A more detailed description of the pathological changes is given in a later section.

*Mortality rate and spleen weights*

The pattern of deaths following intraperitoneal virus inoculation was essentially similar in all infected groups of animals. Fig. 1 shows the mortality distribution of a group of 66 young adult (5–6 weeks old) Swiss mice having an equal number of male and female animals (upper chart). The lower chart in Fig. 1 gives the range of spleen wet weights of another group of 50 mice of the same age which were sacrificed at intervals after inoculation. This chart also includes the spleen weight range of a saline injected control group. Control spleen weights in these animals ranged from 0·25 g. to 0·68 g.

Gross splenic enlargement was detectable within 10 days after virus inoculation and reached its peak at a time corresponding to the maximum death rate. Splenic rupture, which was reported as occurring frequently in Friend's leukaemia (Metcalf, Furth and Buffett, 1959), was seldom seen in this disease. The largest spleen encountered in this group reached a weight of 4·75 g.

The mortality curve shows only one peak (at 30–50 days) again differing from Friend's leukaemia which shows two distinct peaks, one at 10–30 days (associated with splenic rupture) and one at 50–80 days (Metcalf *et al.*, 1959). Over 90 per cent (62 of 66 animals) in this group died within 3 months of inoculation.

*Pathology*

The peripheral blood picture was followed at intervals after inoculation of a group of 25 animals with the leukaemia virus. Haematocrit levels and white cell counts were made at the times indicated on the curves in Fig. 2. Each point

FIG. 1.—Chart snowing mortality (upper portion) and range of spleen wet weights (lower portion)* of Swiss mice inoculated with S37 monocytic leukaemia virus. The numbers of mice represented are 66 and 50 respectively.

* Stippled area—leukaemic mice.
Hatched area—controls.

represents the average of measurements made on 3 different animals. The horizontal dotted lines indicate the range of white cell counts found in a control group of 25 mice injected with saline.

The normal haematocrit level of these animals was 40 to 45 per cent and the inoculated mice showed slightly lowered haematocrits by approximately 20 days after infection. After 2 months, levels were down to 25–30 per cent in many cases.

The total peripheral white count remained steady or slightly low during the first 30 days and then greatly elevated levels were encountered as the disease progressed. In later appearing cases, differential counts showed a marked monocytosis with some erythroblastosis and an increase in lymphocytes. The proportion of erythroblasts appeared to rise steadily, constituting approximately

FIG. 2.—Haematocrit levels and total white cell counts of peripheral blood from 25 S37 monocytic leukaemia injected Swiss mice. Horizontal lines indicate the average normal white cell counts, with the range, in control animals. Each point is the average of values obtained from 3 different animals taken in rotation during the course of the experiment.

6 per cent at 67 days and 24 per cent at 99 days. In the same period the proportion of lymphocytes dropped from 40 to 20 per cent. Polymorphonuclear cells accounted for some 20–30 per cent of the total count while monocytes made up 13–14 per cent and monoblasts 9–12 per cent. The latter resembled the " Friend " cell described as occurring in the blood of mice in the advanced stages of the virus-induced reticulum cell neoplasm of Friend (1957). Fig. 4 shows the typical appearance of these cells which are characterized by a hyperchromatic nucleus, a denser chromatic arrangement than normal and an increased basophilia in the cytoplasm. The nucleus sometimes showed marked variation in shape and size, in contrast to normal monocytes. Cytoplasmic budding, described by Metcalf *et al.* (1959) as occurring in Friend cells, was often noted and resulted in cytoplasmic masses roughly spherical in shape appearing in the blood smears.

Grossly the enlarged spleens were soft with rounded edges and showed haemorrhagic areas and white foci of neoplastic cells. Microscopically lymphocytic elements had decreased and cells of the erythroblastic series increased. Infiltration of the characteristic large monoblastic cells, already described as occurring in the peripheral blood, was widespread (Fig. 5). These cells are assumed to arise from a neoplastic change in the reticular tissue.

Although the liver showed little or no enlargement during the main period of mortality, it frequently did in the later appearing cases. The organ became greyish in colour, friable, with rounded edges and a granular surface. Microscopically there was marked infiltration of the liver cords by round cells which were not at all localized as in extramedullary haematopoiesis (Fig. 6 and 7). These cells were chiefly composed of erythroblasts, lymphocytes and the monoblast type cells already described. Similar infiltration of the thymus, lymph nodes and kidney also occurred in advanced cases.

### DISCUSSION

The disease described in this paper resembles in many respects that described by Friend (1957), especially in the type of abnormal cell predominating in the blood picture in the later stages of the disease. The differences we have noted (e.g. a single instead of double mortality peak and the absence of splenic rupture) are probably not important in differentiating between the two conditions but may simply reflect small differences in response by our Swiss mice which are heterozygous. Friend's leukaemia arose as the result of inoculating a cell-free preparation of Ehrlich ascites cells, and one of the tissues from which the chloroleukaemia of Graffi (1957) was isolated was the Ehrlich carcinoma. Moloney's (1960) lymphoid leukaemia and Franks *et al.* (1957, 1959) monocytic leukaemia both derived from S37. In view of these facts it seems likely that all these virus-induced leukaemias need not be directly related to the tumours from which they were isolated. We agree, therefore, with Moloney (1960) that the virus of S37 monocytic leukaemia like that of his lymphoid leukaemia probably resides in the S37 tumour cells as a " passenger " which happens to favour this milieu. Whether the virus isolated from the leukaemia described here is related to that of Friend (1957) will have to be determined by immunological studies.

We are inclined to regard the characteristic cell found in the peripheral blood and infiltrating the spleen and liver of infected animals as a monoblast and refer to the disease as S37 monocytic leukaemia. As in the case of Friend's disease,

---

### EXPLANATION OF PLATES

Fig. 3.—Mouse dead of S37 monocytic leukaemia approximately 5 months after inoculation. White areas of neoplastic cells in spleen. Spleen, liver, kidney, thymus and lymph nodes all greatly enlarged.

Fig. 4.—Monoblast cells typical of those found in peripheral blood of virus inoculated mice. The upper monoblast seems to be forming a cytoplasmic " bud " and a free " bud " appears beside the lower monoblast. (Wright's Stain—× 1100.)

Fig. 5.—Section through one of the infiltrated areas of an infected spleen showing many monoblasts among the normal erythrogenic cells. (H. & E. × 210.)

Fig. 6.—Section through infected liver in the region of a blood vessel. Monoblasts are evident in the blood and infiltrating the liver sinusoids. (H. & E. × 170.)

Fig. 7.—Neoplastic infiltration of the liver cords of a mouse infected with S37 monocytic leukaemia. (H. & E. × 170.)

3

Bather.

4 5 6 7

Bather.

the erythroblastosis encountered here is probably a secondary effect of the virus, the major cause of death deing due to infiltration of, and interference with, the vital organs by the monoblastic cell. Cytogenetic studies made on these cells are reported by Wakonig-Vaartaja (1960) in a concurrent paper.

### SUMMARY AND CONCLUSIONS

A monocytic leukaemia is described which has resulted from infection by a virus originally isolated from S37 sarcoma tissue and from the spleens of animals surviving S37 sarcoma growth and regression.

Advanced cases of the disease are characterized by greatly enlarged spleen, liver and, in some cases, lymph nodes, thymus and kidney. Splenic enlargement is consistent, and is detectable within 10 days after virus inoculation. Maximum mortality rate occurs between 30 and 50 days after inoculation, and is associated with the extent of splenic enlargement and infiltration with erythroblastic and monoblastic cells.

Erythroblastosis and monoblastosis occur in the peripheral blood late in the disease. The monoblasts resemble the " Friend " cell of Friend's reticulum cell sarcoma and exhibit variation in the shape and size of the nucleus, characteristics of staining and cytoplasmic " budding " described for the Friend cell.

All expenses in connection with this work were borne by the National Cancer Institute of Canada. I should like to thank Dr. R. W. Begg for discussions concerning the pathological observations and Miss Irene Sebastian for her excellent technical assistance.

### REFERENCES

BATHER, R.—(1953) *Brit. J. Cancer*, **7**, 492.
*Idem*, WAKONIG, R., SEBASTIAN, I. AND BEGG, R. W.—(1960) *Proc. Canad. Fed. biol. Sci.*, **3**, 9.
FRANKS, W. R., McGREGOR, A., SHAW, M. M. AND SKUBLICS, J.—(1957) *Cancer Res.*, **17**, 202.—(1959) *Proc. Amer. Ass. Cancer Res.*, **3**, 19.
FRIEND, C.—(1957) *J. exp. Med.*, **105**, 307.
GRAFFI, A.—(1957) *Ann. N.Y. Acad. Sci.*, **68**, 540.
METCALF, D., FURTH, J. AND BUFFETT, R. F.—(1959) *Cancer Res.*, **19**, 52.
MOLONEY, J. B.—(1960) *J. nat. Cancer Inst.*, **24**, 933.
WAKONIG-VAARTAJA, R.—(1961) *Brit. J. Cancer*, **15**, 120.